# Does environmental, social, and governance news coverage affect the cost of equity? A textual analysis of media coverage

**DOI:** 10.3389/fpubh.2025.1509167

**Published:** 2025-04-11

**Authors:** Haixu Yu, Chuanyu Liang, Weiran Wang, Xinglin Liu

**Affiliations:** ^1^School of Economics, Shenzhen Polytechnic University, Shenzhen, China; ^2^Department of Finance, Southern University of Science and Technology, Shenzhen, China; ^3^School of Economics and Management, Tsinghua University, Beijing, China

**Keywords:** ESG performance, cost of equity, news coverage, information asymmetry, media attention

## Abstract

**Introduction:**

Amid growing global environmental challenges and the pursuit of sustainable development, Environmental, Social, and Governance (ESG) has become an important framework for promoting green and responsible business practices. This paper investigates how ESG-related media coverage affects the cost of equity, with a focus on how improved information transparency can influence firms’ financing outcomes.

**Methods:**

We construct an ESG performance index using a machine learning approach, based on ESG-related news text data and the level of media attention received by listed companies. The index is calculated by evaluating the tone (positive or negative) of each firm’s annual ESG news coverage.

**Results:**

The results reveal a significant negative correlation between corporate ESG media coverage and the cost of equity, where more positive coverage is associated with a lower financing cost. Mechanism analysis confirms that stronger ESG performance can reduce equity costs by enhancing the transparency of corporate information. Heterogeneity analysis further shows that this negative relationship is more pronounced in state-owned and large firms.

**Discussion:**

The findings provide empirical evidence for the relationship between ESG news coverage and the cost of equity, validating this link in the context of an emerging market. These results offer new insights into how ESG engagement improves capital market efficiency and supports the broader goals of sustainable and responsible investment.

## 1 Introduction

In recent years, global environmental problems, represented by extreme weather, have become increasingly obvious, offering challenges to human society. It has motivated people to put the concept of sustainable development into reality and to support economic and social development that is greener and less carbon-intensive. China, the world’s second-largest economy, is also actively pursuing sustainable development, and its 2024 report proposes a “dual-carbon” goal that promotes a green transformation of development methods and the continuous enhancement of sustainable development capacity.

Sustainable development philosophy emphasizes economic growth, environmental protection, and social justice (1–3). The concept of sustainable development, as expressed in the ESG framework, corresponds well with the global trend towards green and low-carbon development. The comprehensive indicators comprised by ESG performance also serve as a benchmark for evaluating the effectiveness of related policies. It is increasingly recognized and accepted as a crucial approach to achieving high-quality development globally. ESG performance reflects a company’s ability to manage long-term environmental, social, and governance issues and industry risks (3, 4). The number of ESG disclosures by Chinese listed companies is gradually increasing. According to the most recent CSI ESG statistics, as of April 30, 2024, more than 2,110 A-share listed businesses had published independent sustainability reports, a 16% year-on-year growth, accounting for 39% of all A-share listed companies.

This growing focus on ESG performance is not only driven by investors but also by the significant role of media attention. As an important informal institutional arrangement, media plays an agenda-setting function, reinforcing certain perceptions and generating public expectations and pressures that motivate companies to adopt ESG practices. While much of the existing ESG research has relied on ESG ratings, some scholars have also conducted research on ESG news coverage. Prior research evidence suggests that media coverage of firms’ environmental, social, and governance (ESG) incidents can affect stock prices (5, 6), firm value (7), firm risk (8, 9), and analysts’ forecasts (10), but there is a lack of empirical research on the benefits of ESG news coverage on the capital market. To fill this gap, this paper attempts to explore the following questions: In the presence of ESG investors who focus on non-financial metrics in the financial market, how does a company’s ESG disclosure affect value investors’ access to information and cost of equity?

This study investigates the correlation between the ESG news coverage and equity costs of Chinese A-share-listed companies from 2010 to 2021. We construct an ESG media performance index based on the tone of the media coverage of ESG news about listed companies. When a company actively engages in ESG practices and the media news coverage is more active and positive, the ESG media index will increase if the company receives more media attention. The findings suggest that a higher ESG media index is associated with a decrease in the expense of equity financing. Specifically, for each one standard deviation rise in the ESG media index, the average cost of equity financing decreases by 0.054%. The results remain valid following the robustness test. The mechanism test indicates that firms with high ESG news coverage can reduce their equity financing costs by improving information transparency. Heterogeneity analyses show that this effect is more pronounced in state-owned enterprises as well as in firms with large firm sizes.

This paper makes several contributions. Firstly, we construct an ESG media performance index. The majority of current ESG research concentrates on ESG rating and rating divergence discussions, while fewer studies examine the influence of news coverage on ESG performance. This paper offers additional empirical evidence for ESG research. Secondly, we construct an index of ESG performance under media coverage by analyzing millions of ESG news texts. This index is capable of providing more objective and distinctive corporate information. Thirdly, we find that ESG news coverage can decrease the cost of equity by enhancing the information environment. The findings complete the logical chain of ESG information influencing financing costs. Finally, we consider the internal conditions under which ESG information impacts financing costs and offer targeted recommendations for improving ESG’s role.

## 2 Hypothesis development

The cost of equity is the internal rate of return (or discount rate) on the current market value of a firm, as determined by investors based on the firm’s future cash flows (10). For investors, this rate of return is required to take on the firm’s risk in a specific market situation.

We argue that positive news coverage reduces the cost of equity for two main reasons. Firstly, good ESG performance can enhance a firm’s reputation and attract more investors, which in turn lowers the firm’s cost of equity capital (11). Positive corporate performance in environmental preservation, such as reducing carbon emissions and environmental pollution, can enhance a company’s market image (2, 12). Positive corporate performance in social responsibility, such as labor rights protection, supply chain management, and community involvement, is usually recognized by more investors and consumers (13). Good corporate governance structures, such as efficient boards of directors, transparent financial disclosure, and effective internal controls, can enhance investor confidence (14). Conversely, deficient ESG performance may lead to negative press coverage and increase the cost of financing for the firm. For example, firms may be facing public criticism and regulatory penalties as a result of environmental violations (15). Deficiencies in corporate social responsibility and issues in corporate governance, such as excessive managerial intervention or financial misconduct, can have significant adverse effects on a company’s financial condition when exposed, ultimately leading to an increase in its cost of capital (16, 17).

Secondly, positive ESG news coverage can further reduce the cost of equity by enhancing a firm’s transparency. When firms clearly report on their environmental practices, such as carbon footprint reductions, or social initiatives, like labor rights and diversity efforts, they provide investors with valuable insights that reduce perceived risks (18, 19). Transparent governance practices, including board structure and internal controls, also foster investor confidence (20). On the other hand, a lack of ESG disclosures can lead to mistrust and higher capital costs (21). Therefore, positive ESG disclosure not only enhances investor confidence, but also reduces financing costs by creating a more favorable investment environment. Therefore, we propose hypothesis 1.

*Hypothesis 1: There is a negative correlation between the ESG media performance index and the cost of equity, the higher the media performance index, the lower the cost of equity.*

## 3 Data and methodology

### 3.1 Data

We selected Chinese A-share listed companies from 2010 to 2021 for the research. The ESG news text data used in this study primarily comes from the Datago database, which includes news coverage from third-party media outlets such as financial websites (e.g., Hexun, CICC Online) and financial newspapers (e.g., China Securities Journal, Economic Reference News, People’s Daily). To ensure the comprehensiveness and representativeness of the data, we supplemented the database with manual searches and verification using the Baidu News website. Only news articles directly related to ESG issues (environmental, social, and governance) are retained. We apply natural language processing (NLP) and TF-IDF filtering techniques to extract news articles that contain highly relevant ESG keywords, such as “carbon neutrality,” “corporate social responsibility,” and “board independence.” This ensures that the news articles included in our dataset are genuinely relevant to ESG performance rather than general corporate news.

The financial data was obtained from the Wind database. We exclude firms with special treatment (ST) and financial sectors in the data as well as culling missing data and obtain a total of 13,628 firm-year observations. We conducted winsorization at the 1 and 99% levels for continuous variables to mitigate the impact of outliers and employed clustered regression in regression models. To examine the lead–lag relationship, the sample period for the equity cost indicator was set from 2011 to 2021, while the sample period for company ESG data and control variables was from 2010 to 2020.

### 3.2 Variables

#### 3.2.1 The measure of cost of equity

Cost of equity is a forward-looking indicator based on expected cash flows and is not directly available (22). Following Li et al. (10), we use the price-earnings-growth model (PEG model) proposed by Easton (23) to calculate the cost of equity. The PEG model is calculated as follows:


(1) $$r_{\mathrm{PEG}}=\sqrt{\frac{eps_{2}-eps_{1}}{P_{0}}}$$

where $r_{\mathrm{PEG}}$ is the cost of equity calculated by the model, $P_{0}$ is the price per share at the end of period *t*, $eps_{1}$ is the earnings per share forecast by analysts in period *t + 1*, and $eps_{2}$ is the predicted earnings per share in period *t + 2*.

#### 3.2.2 ESG media index

We construct the ESG performance index based on listed companies’ ESG news text data, as well as the degree of media attention. We obtained all relevant news of listed companies from 2010 to 2021 from the Datago database, supplementing it with manual search and proofreading using the Baidu news website. The ESG media performance index was constructed using a hybrid approach that integrates TF-IDF-based text filtering with a lexicon-based sentiment classification method. ESG-related news articles were first identified using TF-IDF scores to retain highly relevant ESG content. The sentiment of each article was then computed using the Loughran and McDonald (24) financial sentiment dictionary. The firm-year ESG index was derived by aggregating sentiment scores across all firm-specific news, weighted by TF-IDF-based relevance scores. Finally, the ESG media performance index is measured by scoring ESG performance based on the tone (positive or negative) of firm i’s annual news coverage in year *t*. According to An et al. (25), the number of company news signifies the level of media attention. This paper considers the number of news when calculating the ESG performance index, and weights it based on the amount of news. The ESG media index ranges from −1 to 1, with closer to 1 indicating better ESG performance of these firms and closer to −1 indicating poorer ESG performance of these firms. The index differs from traditional ESG ratings in that it is based on news to gather and quantitatively analyze information about a company’s ESG performance.

#### 3.2.3 Control variables

Following El Ghoul et al. (11) and Cook and Luo (26), we selected the following control variables: return on assets (*ROA*), leverage ratio (*Lev*), institutional ownership proportion (*IO*), firm size (*Size*), Tobin’s Q (*TobinQ*), book-to-market ratio (*BM*), revenue growth rate (*Growth*), and board size (*Board*). In the Chinese market, the selected control variables are particularly relevant due to the unique characteristics of China’s financial system, corporate governance, and regulatory environment. Chinese firms primarily rely on bank loans rather than equity financing, making profitability (*ROA*) and leverage (*LEV*) critical factors in determining financing constraints and capital costs. Institutional ownership (*IO*) plays an increasingly significant role, enhances corporate governance and influences market perception. Tobin’s Q and the book-to-market ratio (*BM*) capture firm valuation and investment attractiveness, especially in the Chinese market, where high Tobin’s Q firms (e.g., technology and renewable energy sectors) benefit from government incentives, while high BM firms (e.g., traditional manufacturing industries) face higher risk premiums due to slower ESG compliance adoption. Revenue growth rate (*Growth*) is another critical factor, as China’s economic transition has created volatility in high-growth firms, particularly in innovative industries, leading to fluctuations in their cost of equity. By incorporating these market-specific factors, our study ensures that the control variables effectively capture both firm-level and macroeconomic influences affecting equity financing in China. Table 1 presents the definitions of these variables.

**Table 1:** Variable definitions.

Variables	Symbol	Definition
Dependent variable	*EquityCost*	Cost of equity, calculated using the PEG model.
Explanatory variable	*ESG_index*	ESG media index, calculated based on news coverage of listed companies, ranges from −1 to 1.
Control variables	*ROA*	Return on assets, equal to the ratio of net profit to total assets
	*Lev*	Leverage ratio, equal to the ratio of total liabilities to total assets
	*IO*	Institutional ownership, equal to the ratio of the number of shares held by institutions to the total number of shares outstanding
	*Size*	Firm size, equal to the logarithm of total assets
	*TobinQ*	Tobin’s Q, equal to the ratio of the market capitalization of firms to the replacement cost of firms.
	*BM*	Book-to-market ratio, equal to the ratio of book value to market value
	*Growth*	Growth rate, equal to the growth rate of main operating income
	*Board*	Board size, equal to the number of people on the company’s board of directors.

#### 3.2.4 Model

To examine the impact of ESG media index on equity financing costs, we constructed a regression model. Concurrently, we controlled for both annual fixed effects and industry fixed effects in all regressions. The model is represented by Equation 2:


(2) $$\begin{array}{l} \mathrm{EquityCos}t_{i,t+1}=\beta_{0}+\beta_{1}ESG\_\mathrm{inde}x_{i,t}+\beta_{k}\mathrm{Control}s_{i,t}+\\ \mathrm{Industry}+\mathrm{Year}+\varepsilon_{i,t} \end{array}$$

where $\mathrm{EquityCos}t_{i,t+1}$ is the cost of equity of firm *i* in year *t + 1*, ESG_index is the ESG media performance index of firm *i* in year *t*, $\mathrm{Control}s_{i,t}$ is the control variable, and $\varepsilon_{i,t}$ is the random error term.

## 4 Empirical results

### 4.1 Descriptive statistics

Table 2 reports primary variable descriptive statistics. The *EquityCost* has an average value of 0.108, with a maximum value of 0.241 and a minimum value of 0.025. The standard deviation is 0.040, indicating significant variation among companies, and the cost of equity remains relatively high for a portion of enterprises. For *ESG_index*, the average is 0.372, the maximum is 0.924, the minimum is −0.588, and the standard deviation is 0.327. Control variable distribution is reasonable.

**Table 2:** Descriptive statistical results.

Variable	*N*	Mean	Std. dev	Min	P50	Max
*EquityCost*	13,628	0.108	0.040	0.025	0.104	0.241
*ESG_index*	13,628	0.372	0.327	−0.588	0.430	0.924
*ROA*	13,628	0.042	0.060	−0.217	0.039	0.212
*LEV*	13,628	0.432	0.207	0.007	0.425	1.712
*IO*	13,628	0.393	0.238	0.000	0.400	1.870
*Size*	13,628	22.248	1.289	19.976	22.06	26.246
*TobinQ*	13,628	2.122	2.649	0.609	1.623	8.022
*BM*	13,628	1.032	1.078	0.098	0.675	6.463
*Growth*	13,628	0.245	0.584	−0.545	0.130	4.094
*Board*	13,628	8.634	1.720	0.000	9.000	18.000

### 4.2 Baseline regression

Table 3 displays the baseline regression between the ESG media index and equity costs. Column (1) presents the univariate regression outcomes, with equity cost serving as the dependent variable. The coefficient for *ESG_index* is −0.031, demonstrating significance at the 5% level. Column (2) displays the findings after considering the control variables. The ESG media index coefficient is −0.054, which is statistically significant at 1%. For every 1% increase in the *ESG_index*, there is an average decline of 0.054% in equity costs, confirming that positive media coverage of ESG news helps reduce the cost of equity. This result confirms Hypothesis 1. Furthermore, the lead–lag relationship indicates that ESG information has a long-term effect on equity financing.

**Table 3:** Baseline regression results.

Variable	(1)	(2)
	*EquityCost_t + 1_*	*EquityCost_t + 1_*
*ESG_index_t_*	−0.031^**^ (−2.199)	−0.054^***^ (−4.091)
*ROA_t_*		0.774^***^ (8.306)
*LEV_t_*		0.344^***^ (12.313)
*IO_t_*		−0.111^***^ (−5.717)
*Size_t_*		0.010^*^ (1.848)
*TobinQ_t_*		−0.013^***^ (−2.627)
*BM_t_*		0.179^***^ (5.174)
*Growth_t_*		0.062^***^ (7.240)
*Board_t_*		−0.087^***^ (−3.773)
Constant	1.369^***^ (23.639)	1.150^***^ (9.382)
Observations	13,628	13,628
Adj. *R*^2^	0.181	0.234
Year	Yes	Yes
Industry	Yes	Yes

Robust *t*-statistics are in parentheses. *, **, and *** indicate levels of statistical significance at 10, 5, and 1%, respectively.

### 4.3 Robustness checks

The baseline results indicate that positive media coverage of ESG news helps lower the cost of equity. We conduct robustness tests in order to ensure the reliability of our results and to handle possible issues related to endogeneity, as well as the impact of bias in sample selection. We test the sensitivity of the equity cost measurement indicators, as well as Heckman two-stage tests and Propensity Score Matching (PSM) regression tests.

#### 4.3.1 Sensitivity tests for equity cost

##### 4.3.1.1 Limitations and implications of the PEG model

The PEG model, originally proposed by Easton (23), is widely used in empirical research to estimate the cost of equity. It is particularly useful because it relies on forward-looking earnings forecasts rather than historical data, making it more aligned with investors’ expectations. However, the PEG model has some inherent limitations: First, the model relies on analyst estimates for earnings per share (EPS) in future periods, which may be subject to bias or inaccuracies, especially in markets with limited analyst coverage. Second, the model assumes a stable earnings growth rate, which may not hold in all cases, particularly for firms in volatile industries. Third, any errors in EPS forecasts can directly impact the estimated cost of equity, potentially introducing measurement noise.

Despite these limitations, we believe the PEG model remains a valid and appropriate choice for our study, as it aligns well with prior research on the relationship between ESG factors and equity costs (10, 22). Furthermore, given our study’s focus on ESG news coverage, which influences investor perceptions and future expectations, using a forward-looking model is preferable to backward-looking alternatives, such as historical return-based methods.

##### 4.3.1.2 Results of the sensitivity tests

To validate the robustness of our findings, we use the MPEG model calculations as an alternative variable for the cost of equity, and Equation 3 demonstrates the MPEG model calculations.


(3) $$r_{\mathrm{MPEG}}=\sqrt{\frac{eps_{2}+r_{\mathrm{MPEG}}\times dps_{1}-eps_{1}}{P_{0}}}$$

where $P_{0}$ is the price per share at the end of period *t*, $dps_{1}$ is the dividend per share for a future period, measured using the median of the company’s dividend payout ratio over the past 3 years, $eps_{1}$ is the earnings per share forecast by analysts in period *t + 1*, and $eps_{2}$ is the predicted earnings per share in period *t + 2*.

Table 4 presents the results of the sensitivity tests. The results obtained using the MPEG model remain consistent with those derived from the PEG model. The coefficient of the ESG media index remains negative and statistically significant across both models, confirming that positive ESG news coverage is associated with a lower cost of equity. The magnitude of the effect is slightly larger in the MPEG model (−0.064) compared to the PEG model (−0.054), which aligns with expectations since the MPEG model accounts for the impact of dividends on investor valuation. The statistical significance of the results (at the 1% level) further reinforces the robustness of our findings.

**Table 4:** Sensitivity tests for cost of equity.

Variable	(1)	(2)
	*MPEG_t + 1_*	*MPEG_t + 1_*
*ESG_index_t_*	−0.039^*^ (−1.686)	−0.064^***^ (−2.712)
*ROA_t_*		1.736^***^ (11.166)
*LEV_t_*		0.241^***^ (4.526)
*IO_t_*		−0.128^**^ (−2.516)
*Size_t_*		0.024^**^ (2.382)
*TobinQ_t_*		−0.009 (−0.585)
*BM_t_*		0.459^***^ (7.651)
*Growth_t_*		0.053 (1.332)
*Board_t_*		−0.131^***^ (−2.783)
Constant	1.330^***^ (86.859)	1.092^***^ (5.866)
Observations	13,628	13,628
Adj. *R*^2^	0.021	0.098
Year	Yes	Yes
Industry	Yes	Yes

Robust *t*-statistics are in parentheses. *, **, and *** indicate levels of statistical significance at 10, 5, and 1%, respectively.

#### 4.3.2 Sample selection bias

Furthermore, we conducted robustness checks using Heckman two-stage tests and PSM regression. The results are displayed in Table 5. Column (1)–(2) report the results of Heckman two-stage regression. In the first stage, Inverse Mills Ratio (IMR) is calculated and the variable is controlled in the second stage. The results show a negative and significant relationship between the ESG media index and cost of equity. Column (3)–(4) report the results of the PSM regression. We first conducted a regression analysis to examine the relationship between the *ESG_index* and the control variables. Additionally, we calculated propensity scores for each variable. Based on these scores, we paired the control and treatment groups using 1:1 closest neighbor matching without replacement and matched 4,191 samples using *EquityCost*. Column (3) indicate that the PSM approach operates since the experimental and control groups have balanced control variables. The coefficient for the *ESG_index* in Column (4) is −0.049, indicating a negative association at the 1% significance level. The results are consistent with the baseline regression analysis, indicating that there was no sample selection bias.

**Table 5:** Results from Heckman two-stage test and PSM regression.

Variable	Heckman two-stage		PSM regression	
	(1)	(2)	(3)	(4)
	*Treat*	*EquityCost _t + 1_*	*ESG_index_t_*	*EquityCost_t + 1_*
*ESG_index_t_*		−0.084^***^ (−4.162)		−0.049^***^ (−2.644)
*ROA_t_*	2.082^***^ (7.212)	1.297^***^ (8.516)	3.780^***^ (7.340)	0.436^***^ (2.968)
*LEV_t_*	−0.037(−0.437)	0.536^***^ (21.471)	−0.089 (−0.590)	0.279^***^ (6.234)
*IO_t_*	0.236^***^ (3.724)	−0.109^***^ (−4.712)	0.428^***^ (3.820)	−0.122^***^ (−3.814)
*Size_t_*	0.260^***^ (15.871)	−0.029^**^ (−2.028)	0.465^***^ (15.680)	0.034^***^ (3.610)
*TobinQ_t_*	−0.032^*^ (−1.673)	−0.009^*^ (−1.645)	−0.053 (−1.580)	−0.015 (−1.517)
*BM_t_*	−0.695^***^ (−6.328)	0.327^***^ (6.504)	−1.211^***^ (−6.240)	0.106^*^ (1.769)
*Growth_t_*	0.059^*^ (1.700)	0.090^***^ (8.630)	0.105 (1.640)	0.088^***^ (5.741)
*Board_t_*	0.132^*^ (1.915)	−0.092^***^ (−4.443)	0.228^**^ (1.860)	−0.110^***^ (−3.076)
Constant	−4.884^***^ (−14.283)	1.568^***^ (4.157)	−9.846^***^ (−14.290)	0.804^***^ (3.938)
Observations	13,628	13,628	13,628	4,191
Year	Yes	Yes	Yes	Yes
Industry	Yes	Yes	Yes	Yes
IMR	0.122^*^ (0.069)			

Robust t-statistics are in parentheses. *, **, and *** indicate levels of statistical significance at 10, 5, and 1%, respectively.

## 5 Further analysis

### 5.1 Mechanism test

#### 5.1.1 The impact of media attention in the mechanism test

In our previous regression analysis, we confirmed that there is a negative association between the ESG media index and equity costs. This section will examine how the ESG coverage affects equity costs. Media attention plays a crucial role in reducing information asymmetry. Information asymmetry refers to the disparity of information between firms and their investors or other stakeholders, which can lead to market inefficiencies and increased capital costs. When the media extensively covers a firm’s operations, governance, and strategic decisions, it enhances information transparency, allowing investors to better understand the firm’s true state, thereby reducing information asymmetry (27). Additionally, media attention helps improve a firm’s public image and reputation, further reducing uncertainty for market participants (28). Particularly in the context of ESG issues, positive media coverage can significantly enhance the firm’s credibility and transparency in the eyes of the public, thereby reducing uncertainties surrounding the firm’s long-term prospects and risks and lowering the cost of equity financing (29, 30). For example, media coverage focusing on a firm’s ESG performance not only compensates for the limitations of official ESG ratings but also aids external stakeholders in accessing more comprehensive and timely information about the company (31). Recent studies indicate that social media and news platforms have also increasingly played a significant role in disseminating corporate ESG performance and financial information. The broad reach and speed of information dissemination on these platforms have further amplified transparency, thereby reducing information asymmetry (32). Therefore, media attention has a significant impact on reducing information asymmetry, enabling investors to make more informed decisions by providing them with greater access to information.

Furthermore, previous studies suggest that effective corporate governance, especially comprehensive information disclosure, can mitigate agency conflicts and information asymmetry between management and investors, lowering capital costs (33–35). Information asymmetry constrains investor trading behavior, but detailed disclosure improves stock liquidity (33, 36). High stock liquidity often lowers company financing costs. The media helps investors, shareholders, and other stakeholders in making decisions by disseminating critical information such as firm financial data, management decisions, and strategy (28). Increased transparency of information helps to reduce information asymmetry, improving the operational efficiency of markets (27, 37). Therefore, we suggest that disclosure of ESG information improves transparency and reduces the cost of equity for firms. Information asymmetry is identified as a key influencing mechanism.

#### 5.1.2 Results of the mechanism test

To empirically test this mechanism, we conducted a two-stage regression analysis. In the first stage, we regressed *ESG_index* on information asymmetry (*ASY*). We construct an indicator of information asymmetry (*ASY*) using the liquidity indicator (*LR*) (38), the illiquidity ratio (*ILLIQ*) (39), and the earnings reversal indicator (*GAM*) (40). Detailed calculations are given in Appendix A. In this stage, ESG news coverage was included as the key explanatory variable to examine its impact on reducing information asymmetry. The regression model is shown as Equation 4.


(4) $$\begin{array}{l} ASY_{i,t+1}=\beta_{0}+\beta_{1}ESG\_\mathrm{inde}x_{i,t}+\beta_{k}\mathrm{Control}s_{i,t}+\\ \mathrm{Industry}+\mathrm{Year}+\varepsilon_{i,t} \end{array}$$

where $ASY_{i,t+1}$ represents the level of information asymmetry in year *t* + 1, measured by liquidity and earnings reversal indicators. $ESG\_\mathrm{inde}x_{i,t}$ is the ESG media performance index for firm 𝑖 in year 𝑡.

In the second stage, we regressed information asymmetry (*ASY*) on the cost of equity (*EquityCost*) to analyze how changes in information asymmetry influence a firm’s equity financing costs. This two-stage approach allowed us to isolate the effect of media attention on information asymmetry, and subsequently, assess its indirect impact on the cost of equity. By examining the relationship between ESG news coverage, information asymmetry, and equity costs, we were able to identify the underlying mechanism through which media attention reduces financing costs. The regression model is shown as Equation 5.


(5) $$\begin{array}{l} \mathrm{EquityCos}t_{i,t+1}=\beta_{0}+\beta_{1}ASY_{i,t}+\beta_{k}\mathrm{Control}s_{i,t}+\\ \mathrm{Industry}+\mathrm{Year}+\varepsilon_{i,t} \end{array}$$

where $\mathrm{EquityCos}t_{i,t+1}$ is the cost of equity of firm *i* in year *t + 1*, $ASY_{i,t}$ represents the level of information asymmetry in year *t*.

Table 6 shows the mechanism test results. Column (1) shows the ESG media index and information asymmetry (*ASY*) regression results. The *ESG_index* coefficient is −0.044, significant at 1%, demonstrating that firm information asymmetry decreases with ESG media index. Column (2) investigates the correlation between information asymmetry and equity cost. The findings indicate that the *ASY* is 0.073, significant at 1%. ESG information improves the firm’s information environment and reduces information asymmetry, lowering financing costs.

**Table 6:** Results of mechanism tests.

Variable	(1)	(2)
	*ASY_t + 1_*	*EquityCost_t + 1_*
*ESG_index_t_*	−0.044^***^ (−5.786)	
*ASY_t_*		0.073^***^ (5.200)
*ROA_t_*	0.001 (0.019)	0.755^***^ (8.099)
*LEV_t_*	0.334^***^ (16.282)	0.328^***^ (11.617)
*IO_t_*	0.445^***^ (25.872)	−0.147^***^ (−7.286)
*Size_t_*	−0.394^***^ (−48.182)	0.035^***^ (4.662)
*TobinQ_t_*	−0.039^***^ (−6.563)	−0.012^**^ (−2.259)
*BM_t_*	1.167^***^ (37.434)	0.104^***^ (2.818)
*Growth_t_*	0.017^***^ (3.027)	0.059^***^ (6.818)
*Board_t_*	0.034^*^ (1.660)	−0.087^***^ (−3.732)
Constant	7.475^***^ (44.928)	0.659^***^ (4.234)
Observations	13,628	13,628
Adj. *R*^2^	0.593	0.235
Year	Yes	Yes
Industry	Yes	Yes

Robust t-statistics are in parentheses. *, **, and *** indicate levels of statistical significance at 10, 5, and 1%, respectively.

### 5.2 Heterogeneity analysis

State-owned enterprises (SOEs) are crucial for China’s economic development, especially in key and critical sectors. They also represent the government in providing goods and services that private enterprises are unable or unwilling to offer. Generally, SOEs are defined by their substantial asset sizes, robust operational continuity, and strong ability to repay debts. SOEs have a comparative advantage in financing compared to non-SOEs. They have easier access to lower-cost financing with longer repayment periods.

Based on ownership, we divide the sample into SOE and non-SOE groups. Columns (1)–(2) of Table 7 report the impact of differences in ownership type. The SOE group’s *ESG_index* coefficient is −0.065, significant at 1%. The coefficient for the non-SOE group is −0.013, which is not significant. The results of these studies suggest that ESG media coverage is significant for state-owned enterprises. Therefore, changes in ESG media indices reinforce the positive image of SOEs and have a greater impact on reducing the cost of equity. ESG information complements nonfinancial information, provides creditors with greater insight, and reduces the cost of equity.

**Table 7:** The effect of ownership type.

Variable	Ownership type		Firm size	
	(1) SOE	(2) Non-SOE	(3) Large	(4) Small
*ESG_index_t_*	−0.065^***^ (−2.720)	−0.013 (−0.842)	−0.078^***^ (−3.850)	−0.027^*^ (−1.646)
*ASY_t_*	1.113^***^ (6.726)	0.522^***^ (4.687)	0.994^***^ (7.177)	0.522^***^ (4.318)
*ROA_t_*	0.419^***^ (8.742)	0.312^***^ (9.018)	0.406^***^ (9.822)	0.226^***^ (5.959)
*LEV_t_*	−0.044 (−1.259)	−0.040^*^ (−1.696)	−0.136^***^ (−5.231)	−0.058^**^ (−2.076)
*IO_t_*	0.014^*^ (1.660)	0.011 (1.511)	0.001 (0.119)	0.028^**^ (2.187)
*Size_t_*	−0.018^**^ (−2.034)	−0.008 (−1.253)	−0.021^**^ (−2.422)	−0.013^*^ (−1.854)
*TobinQ_t_*	0.212^***^ (3.807)	0.222^***^ (4.924)	0.188^***^ (3.962)	0.139^**^ (2.532)
*BM_t_*	0.026^*^ (1.836)	0.067^***^ (6.355)	0.043^***^ (3.870)	0.091^***^ (7.008)
*Growth_t_*	−0.073^*^ (−1.961)	−0.033 (−1.164)	−0.078^**^ (−2.437)	−0.095^***^ (−3.153)
Constant	0.887^***^ (4.640)	1.057^***^ (6.400)	1.359^***^ (7.473)	0.816^***^ (3.108)
Observations	5,380	8,014	7,814	5,814
Adj. *R*^2^	0.272	0.234	0.255	0.190
Year	Yes	Yes	Yes	Yes
Industry	Yes	Yes	Yes	Yes

Robust *t*-statistics are in parentheses. *, **, and *** indicate levels of statistical significance at 10, 5, and 1%, respectively.

Furthermore, firm size tends to be an important factor affecting the cost of corporate finance. Large firms tend to have a scale effect and are able to obtain funds at a lower cost of capital, whereas small firms tend to demand a higher rate of return from creditors as well as investors due to the limitations of firm size. We divide the sample into large and small firm groups based on the median firm size per year. Columns (3)–(4) of Table 7 demonstrate the effect of differences in firm size on the cost of equity. The results show that the coefficient on *ESG_index* for the large firm group is −0.078, which is significant at the 1% level. The coefficient of −0.027 for the small firm group is significant at the 10% level. The results show that positive ESG news coverage has a more significant effect on reducing the cost of capital in large firms. There are several reasons why the impact of ESG performance is more significant for large firms. First, large firms often have more established reputations and greater access to capital markets, making them more susceptible to investor perceptions regarding ESG factors (41). Additionally, large firms are more likely to be scrutinized by institutional investors who place greater emphasis on ESG performance when making investment decisions (42). Finally, large firms have the resources to implement and report comprehensive ESG strategies, which can further enhance their credibility and appeal to sustainability-conscious investors (16).

## 6 Discussion

Our findings confirm that the ESG media index plays a critical role in shaping corporate financing conditions by improving transparency and investor confidence. To ensure that this relationship is not driven by other firm characteristics, we control for ROA, leverage, firm size, Tobin Q and other variables, following previous literature (43). These controls allow us to better isolate the impact of ESG media coverage on equity costs, reinforcing the robustness of our findings. Even after controlling for these factors, ESG media coverage remains a significant determinant of financing conditions, influencing firms’ risk perception and cost of capital. Firms with greater ESG index are perceived as less risky, leading to lower risk premiums and improved access to capital. This effect is particularly pronounced in firms with stronger governance mechanisms, such as those with larger boards, where enhanced oversight amplifies ESG effectiveness. Similarly, firms with higher market valuations (Tobin’s Q) and lower book-to-market ratios benefit more from ESG transparency, as their valuations rely more on investor sentiment. Moreover, state-owned enterprises (SOEs) experience greater reductions in financing costs through ESG media coverage, likely due to institutional credibility and stronger regulatory oversight. Conversely, while high profitability, revenue growth, and leverage are typically associated with higher equity costs, ESG transparency helps mitigate these concerns by reinforcing financial stability and credibility. These findings highlight ESG transparency as not only a governance mechanism but also a strategic financial asset that enhances market confidence and lowers financing costs.

A key mechanism through which ESG media coverage reduces the cost of equity is enhancing corporate transparency and mitigating information asymmetry. Firms with strong ESG transparency provide more reliable and accessible information, reducing uncertainty and strengthening investor confidence, which aligns with prior research on disclosure and capital costs (33–35). Additionally, ESG transparency improves market liquidity, as greater disclosure attracts investors, lowers transaction costs, and reduces price volatility, facilitating equity financing. In contrast, firms with weaker ESG communication face higher risk premiums and lower investor appeal due to reduced liquidity and greater price fluctuations. These findings underscore ESG transparency as not just a governance tool but a key determinant of financial stability and valuation, reinforcing its increasing importance in investment decisions (36).

To ensure the robustness of our findings, we conducted sensitivity analysis and selection bias tests, confirming that the observed effect of ESG media coverage on reducing the cost of equity is not driven by model specifications or sample selection. When we replaced the PEG model with the MPEG model, the results remained consistent, indicating that our findings hold regardless of the cost of equity estimation method. Additionally, to address potential selection bias—where firms with greater ESG media coverage may inherently possess better governance or financial health—we employed the Heckman two-stage model and Propensity Score Matching (44). Both approaches reaffirm that ESG transparency remains significantly associated with lower equity costs, reinforcing the validity of our conclusions. Further analysis reveals that the benefits of ESG media coverage in reducing financing costs are more pronounced in state-owned enterprises (SOEs) and large firms, suggesting that firms with greater investor scrutiny and institutional credibility gain more from ESG transparency. These findings emphasize that ESG disclosure is a robust determinant of equity financing costs across different firm types and market conditions.

## 7 Conclusion

This paper examines the impact of ESG news coverage on the cost of capital. As a key informal institutional mechanism, the media plays an agenda-setting role, shaping public perceptions and generating expectations that drive companies to adopt ESG practices. According to stakeholder theory, companies engaged in ESG activities enhance their ability to manage environmental, social, reputational, operational, and regulatory risks, which in turn helps reduce their equity costs.

The results demonstrate that companies with more positive ESG media coverage (higher ESG media index) have a lower cost of equity. This finding remains applicable even after conducting sensitivity tests and robustness checks. Mechanism tests suggest that ESG news coverage reduces financing costs by improving the information environment and decreasing information asymmetry. Heterogeneity tests indicate that this effect is more pronounced in government-owned and large firms. Firms that take part in ESG construction can reinforce their public image, build their reputational capital, and lower their financing costs. This research expands our understanding of the factors affecting equity costs and complements studies on the impact of corporate participation in ESG governance. It contributes to promoting a correct understanding and better construction of ESG in enterprises.

Based on the findings of this study, the following policy recommendations can be made to lower corporate financing costs through improved ESG practices. First, policymakers should strengthen ESG disclosure regulations to enhance information transparency and reduce information asymmetry. Second, media outlets should be encouraged to highlight positive ESG practices and be incentivized to promote accurate and impactful ESG reporting. Third, firms should enhance their corporate governance structures by integrating ESG principles into their operations, with a particular focus on the establishment of sustainability committees and mechanisms for executive accountability. Finally, firms and policymakers should collaborate to advance green innovation and investment, aligning corporate strategies with environmental sustainability goals to effectively reduce financing costs and achieve a dual benefit of financial and societal value.

This study provides empirical evidence supporting the relationship between ESG news coverage and the cost of equity, validating this link within the context of emerging market. Future research could extend this analysis to developed markets, such as the US and European stock market, to examine whether similar relationships hold under different institutional settings. Additionally, as ESG regulations and media influence evolve, future studies could explore how this relationship changes over time and across different economic cycles.

## Data Availability

The raw data supporting the conclusions of this article will be made available by the authors, without undue reservation.

## Appendix A. Information asymmetry construction method

We construct an indicator of information asymmetry (*ASY*) using the liquidity indicator (*LR*) (38), the illiquidity ratio (*ILLIQ*) (39), and the earnings reversal indicator (*GAM*) (40).

Firstly, the illiquidity ratio indicator (*LR*) is calculated as shown in Equation A1:

(A1)
$$LR_{i,t}=-\frac{1}{D_{i,t}}\sum_{k=1}^{D_{i,t}}\sqrt{\frac{V_{i,t}\left(k\right)}{\left|r_{i,t}\left(k\right)\right|}}\text{,}$$

where $r_{i,t}\left(k\right)$ is the stock return of firm *i* on the *k-*th trading day in year *t*, $V_{i,t}\left(k\right)$ is the trading volume of firm *i* on the *k-*th trading day in year *t*, and $D_{i,t}$ is the number of trading days.

Secondly, we calculate the liquidity ratio. A higher *ILLIQ* suggests more company information asymmetry. Specifically, the calculation of the *ILLIQ* is outlined in Equation A2:

(A2)
$$\mathrm{ILLI}Q_{i,t}=\frac{1}{D_{i,t}}\sum_{k=1}^{D_{i,t}}\sqrt{\frac{\left|r_{i,t}\left(k\right)\right|}{V_{i,t}\left(k\right)}}\text{,}$$

where $r_{i,t}\left(k\right)$ is the stock return of firm *i* on the *k-*th trading day in year *t*, $V_{i,t}\left(k\right)$ is the trading volume of firm *i* on the *k-*th trading day in year *t*, and $D_{i,t}$ is the number of trading days.

Finally, the earnings reversal indicator (*GAM*) is calculated to measure stock market liquidity as shown in Equations A3–A5.

(A3)
$$GAM_{i,t}=|\gamma_{i,t}|$$

where $\gamma_{i,t}$ is estimated from Equation A4, $r_{i,t}^{e}\left(k\right)$ is the excess return.

(A4)
$$r_{i,t}^{e}\left(k\right)=\theta_{i,t}+\varphi_{i,t}r_{i,t}\left(k-1\right)+\gamma_{i,t}V_{i,t}\left(k-1\right)\mathrm{sign}\left[r_{i,t}^{e}\left(k-1\right)\right]+\varepsilon_{i,t}\left(k\right)$$

where $r_{i,t}^{e}\left(k\right)$ , is calculated from Equation A5:

(A5)
$$r_{i,t}^{e}\left(k\right)=r_{i,t}\left(k\right)-r_{m,t}\left(k\right)$$

where $r_{m,t}\left(k\right)$ is the market return weighted by market capitalization outstanding.

The principal component analysis (PCA) was used to fit the information asymmetry indicator to the variables *IR*, *ILLIQ*, and *GAM*. The *ASY* consisted of the asymmetric information elements present in all three components.
